# Detrimental Effects of Testosterone Addition to Estrogen Therapy Involve Cytochrome P-450-Induced 20-HETE Synthesis in Aorta of Ovariectomized Spontaneously Hypertensive Rat (SHR), a Model of Postmenopausal Hypertension

**DOI:** 10.3389/fphys.2018.00490

**Published:** 2018-05-08

**Authors:** Tiago J. Costa, Graziela S. Ceravolo, Cinthya Echem, Carolina M. Hashimoto, Beatriz P. Costa, Rosangela A. Santos-Eichler, Maria Aparecida Oliveira, Francesc Jiménez-Altayó, Eliana H. Akamine, Ana Paula Dantas, Maria Helena C. Carvalho

**Affiliations:** ^1^Department of Pharmacology, Institute of Biomedical Sciences, University of São Paulo, São Paulo, Brazil; ^2^Facultat de Medicina, Departament de Farmacologia, Terapèutica i Toxicologia, Institut de Neurociències, Universitat Autònoma de Barcelona, Bellaterra, Spain; ^3^Group of Atherosclerosis and Coronary Disease, Institut Clinic del Torax, Institut d’Investigacions Biomédiques August Pi I Sunyer, Barcelona, Spain; ^4^Department of Physiological Sciences, State University of Londrina, Londrina, Brazil

**Keywords:** testosterone and estrogen treatment, spontaneously hypertensive rat (SHR), cardiovascular disease, vascular reactivity, ROS generation, cytochrome P-450 pathways, 20-HETE

## Abstract

Postmenopausal period has been associated to different symptoms such as hot flashes, vulvovaginal atrophy, hypoactive sexual desire disorder (HSDD) and others. Clinical studies have described postmenopausal women presenting HSDD can benefit from the association of testosterone to conventional hormonal therapy. Testosterone has been linked to development of cardiovascular diseases including hypertension and it also increases cytochrome *P*-450-induced 20-HETE synthesis which in turn results in vascular dysfunction. However, the effect of testosterone plus estrogen in the cardiovascular system is still very poorly studied. The aim of the present study is to evaluate the role of cytochrome *P*-450 pathway in a postmenopausal hypertensive female treated with testosterone plus estrogen. For that, hypertensive ovariectomized rats (OVX-SHR) were used as a model of postmenopausal hypertension and four groups were created: SHAM-operated (SHAM), ovariectomized SHR (OVX), OVX treated for 15 days with conjugated equine estrogens [(CEE) 9.6 μg/Kg/day/po] or CEE associated to testosterone [(CEE+T) 2.85 mg/kg/weekly/im]. Phenylephrine-induced contraction and generation of reactive oxygen species (ROS) were markedly increased in aortic rings from OVX-SHR compared to SHAM rats which were restored by CEE treatment. On the other hand, CEE+T abolished vascular effects by CEE and augmented both systolic and diastolic blood pressure of SHR. Treatment of aortic rings with the CYP/20-HETE synthesis inhibitor HET0016 (1 μM) reduced phenylephrine hyperreactivity and the augmented ROS generation in the CEE+T group. These results are paralleled by the increased CYP4F3 protein expression and activity in aortas of CEE+T. In conclusion, we showed that association of testosterone to estrogen therapy produces detrimental effects in cardiovascular system of ovariectomized hypertensive females via CYP4F3/20-HETE pathway. Therefore, our findings support the standpoint that the CYP/20-HETE pathway is an important therapeutic target for the prevention of cardiovascular disease in menopausal women in the presence of high levels of testosterone.

## Introduction

The postmenopausal period has been associated to reduction of plasma estrogen levels and different symptoms such as hot flashes, vulvovaginal atrophy, hypoactive sexual desire disorder (HSDD) and others. At the moment, estrogen therapy is the most effective intervention for hot flashes and improves vulvovaginal atrophy symptoms (Leiblum et al., 2006; Hayes et al., 2007; Shifren et al., 2008; Takahashi and Johnson, 2015), but not HSDD.

Hypoactive sexual desire disorder affects 40% of postmenopausal women (Hayes et al., 2007; Shifren et al., 2008) and, for that reason, testosterone has been used with increasing frequency to treat this condition (Dennerstein et al., 2006; Leiblum et al., 2006; Achilli et al., 2016; Shifren and Davis, 2017). However, little is known how this therapy affects the cardiovascular system of menopausal women. Elevated androgen concentrations in natural menopause and polycystic ovary syndrome have been associated with higher incidence of cardiovascular disease (Rexrode et al., 2003; Yanes and Reckelhoff, 2011; Daan et al., 2015). We recently described that testosterone treatment at clinical dose increases blood pressure and aggravates vascular dysfunction in a rat model of hypertensive menopause (Costa et al., 2015).

Among mechanisms to explain the detrimental effects of testosterone on cardiovascular disease, direct actions in the vasculature have been reported such as modulation of cytochrome P-450, cyclooxygenase products, oxidative stress and 20-HETE synthesis (Wu and Schwartzman, 2011; Lopes et al., 2012; Tostes et al., 2015). Cytochrome P450 enzymes of the 4A (CYP4A) and 4F (CYP4F) subfamilies catalyze the ω-hydroxylation of arachidonic acid and produce epoxyeicosatrienoic acids (EETs) and dihydroxyeicosatetraenoic acids (DiHETEs), such as 20-hydroxyeicosatetraenoic acid (20-HETE) (Capdevila et al., 1981; Morrison and Pascoe, 1981; Oliw et al., 1981). Sex-dependent expression and regulation by androgens has been a key feature of CYP4A enzymes (Holla et al., 2001; Yanes et al., 2011; Wu et al., 2013). In fact, CYP4A-derived 20-HETE activity has been increased in either cerebral microvessels from aged female spontaneously hypertensive rats (SHR) (Yanes et al., 2011) or renal microvessels from female normotensive Sprague-Dawley rats with dihydrotestosterone supplementation (Dalmasso et al., 2016).

20-HETE contributes to the regulation of vascular function (Imig, 2016), mainly in the microvasculature (Harder et al., 1994). In those vessels, 20-HETE has been described as a potent vasoconstrictor that induces vascular smooth muscle cell depolarization through inhibition of potassium channel (*K*_Ca_), protein kinase C (PKC) (Lange et al., 1997), Rho Kinase activation (Randriamboavonjy et al., 2003). 20-HETE also increases sensitization of the contractile apparatus to Ca^2+^ (Randriamboavonjy et al., 2003, 2005), inhibits Na^+^-K^+^-ATPase (Ominato et al., 1996; Nowicki et al., 1997), as well as impairs the NO-dependent vasodilatation (Alonso-Galicia et al., 1999; Sun et al., 2000; López et al., 2001) and cGMP/NO-independent vasodilatation (López et al., 2001). Besides 20-HETE may play a central role in vascular remodeling by inducing vascular smooth muscle cell growth, proliferation and differentiation via calcium/calmodulin dependent protein kinase II, leading to activation of mitogen-activated protein kinase signaling pathway (Muthalif et al., 1998). However, as far as we know, the role of 20-HETE in large vessel function is not completely understood.

Recently, increased 20-HETE levels have been related to the development of cardiovascular disease, such as stroke (Huang et al., 2016; Yi et al., 2016a), coronary heart disease (Miyata and Roman, 2005), arterial dysfunction (Berezan et al., 2008) atherosclerosis (Yi et al., 2016b,c) and hypertension (Williams et al., 2010). Postmenopausal women are at greater risk to suffer from these diseases, and testosterone could further increase this risk. In this regard, in the present study we determined the role of a clinically relevant dose of testosterone associated to estrogen conventional therapy in vascular function of aorta of ovariectomized SHR, a model of postmenopausal hypertension, and how 20-HETE could contribute to effects induced by testosterone.

## Materials and Methods

### Animal Model

Female spontaneously hypertensive rats (SHR) were obtained from the breeding stock of the Institute of Biomedical Sciences of the University of São Paulo (ICB-USP) and maintained in a temperature-controlled room on a 12-h light/dark cycle, 60% humidity and standard rat chow and water *ad libitum*. This study was approved by the Institutional Animal Ethics Committee of the ICB-USP (Protocol 145, page 95, book 2. 06.12.2013), following the Guide for the Care and Use of Laboratory Animals published by the US National Institute of Health (NIH Publication No. 85-23, revised 1996).

### Ovariectomy, Estrogen, and Testosterone Treatment

Rats were ovariectomized (OVX) or SHAM-operated (SHAM) at 12 weeks of age under with a mixture of ketamine (113 mg/kg) and xylazine (7.4 mg/kg). Thirty days after ovariectomy, a group of OVX rats was treated during 15 days with a solution of conjugated equine estrogens (CEE) (Premarin^®^, 9.6 μg/Kg/day; p.o.) by gavage as previously described (Ceravolo et al., 2012; Araujo et al., 2017). Another OVX group received CEE (9.6 μg/Kg/day) associated with testosterone cypionate (2.85 mg/kg/weekly, im; CEE+T) as reported (Ramos et al., 2007; Costa et al., 2015). At the end of the treatment period, rats were anesthetized (sodium thiopental, 40 mg/Kg; i.p.), blood samples were collected from the abdominal aorta and then centrifuged to separate the serum. Serum levels of estrogen and testosterone were determined by a commercial ELISA kit (Cayman Chemical, United States).

### Arterial Blood Pressure Measurement

Female rats were anasthetized with ketamine (113 mg/Kg; i.p.) and xylazine (7.4 mg/Kg; i.p.), a heparinized polyethylene catheter was inserted into the right carotid artery and the catheter was exteriorized in the mid-scapular region. After a 24-h recovery period, systolic and diastolic blood pressures were measured in conscious animals by a pressure transducer (Deltran DPT-100, Utah Medical Products, United States) and recorded using an interface and software for computer data acquisition (PowerLab, ADInstruments, Melbourne, VIC, Australia).

### Vascular Reactivity

Endothelium-intact segments (4 mm) of descending thoracic aorta were carefully dissected to remove excess of fat and connective tissue in ice-cold Krebs solution (in mM: 118 NaCl, 4.7 KCl, 25 NaHCO_3_, 2.5 CaCl_2_-2H_2_O, 1.2 KH_2_PO_4_, 1.2 MgSO_4_-7H_2_O, 11 glucose and 0.01 EDTA), and gassed with 95% O_2_ and 5% CO_2_. Two stainless steel hooks were inserted into the lumen of the vessel and set up in an organ bath for measurement of isometric contractile force. The vessels were prepared essentially as previously described (Carvalho et al., 1987). Thoracic aorta segments were submitted to a tension of 1.5 g (Ceravolo et al., 2007). After a 60-min equilibration period, thoracic aortic rings were initially exposed to 90 mM KCl to determine the functional integrity of smooth muscle cells. Contractile responses mediated by α_1_-adrenoceptor stimulation were studied by evaluating cumulative concentration-response curves to phenylephrine (Phenyl: 0.1 nM to 10 μM). In another series of experiments, the influence of 20-HETE on Phenyl responses was evaluated by adding *N*-hydroxy-N′-(4-butyl- 2-methylphenyl)-formamidine (HET0016 – 1 μM; Cayman Chemical, United States – product number 75780), an inhibitor of the CYP4A and CYP4F, to the organ bath 30 min before the concentration-response curves to Phenyl. HET0016 was diluted in DMSO:PBS (pH 7,2; 1:1) solution, and the concentration was chosen on the basis of previous studies (Yousif and Benter, 2010). Similar concentration of DMSO was used in untreated (Control) arteries.

### Detection of Reactive Oxygen Species (ROS) Generation in Aortic Sections

Vascular ROS detection was determined *in situ* in thoracic aortic sections by dihydroethidium (DHE). Before aortas were frozen in freezing medium, they were incubated with HET0016 (1 μM) or vehicle (basal group) for 30 min in Krebs solution at 37°C. Cross sections of aorta (10 μm) were incubated in a light-protected and humidified chamber (37°C, 30 min) with DHE (5 μM) and fluorescence was detected with a 585–590 nm long-pass filter, under a microscope (Nikon, Japan) with a 40× objective lens coupled to a digital camera. Fluorescent images were recorded and analyzed by measuring the mean optical density of the fluorescence using an imaging software (Image J, NIH, United States). Fluorescence was evaluated in each image at least in four locations and normalized by the area.

### Immunoblot Analysis

Frozen thoracic aorta was homogenized in RIPA lysis buffer (Thermo Fisher Scientific) [50 mmol/L of Tris/HCl (pH 7.4), 1% Igepal, 0.25% sodium deoxycholate, 150 mM NaCl, 1 mM EDTA, 2 μg/ml protease inhibitor] and centrifuged at 14000 *g* for 30 min at 4°C. Equal amount of protein (30 μg) from each aorta was resolved by SDS-PAGE on 4–12% gels and electroblotted onto nitrocellulose membrane. Membranes were incubated overnight at 4°C with 1:1000 dilution of polyclonal CYP4A1 and CYP4F3 antibodies (Biorbyt, Cambridge, United Kingdom). After that, membranes were incubated for 1 h with a 1:2000 dilution of horseradish peroxidase-labeled goat anti-rabbit secondary antibody (Thermo Scientific, Waltham, MA, United States) and chemiluminescent signal was visualized by ImageQuant LAS 4000 imaging system (GE Headquarter Life Science, Marlborough, MA, United States). Densitometric analyses of immunoblots were performed using an imaging software (Image J, NIH, United States), and CYP4A1 and CYP4F3 band densities were normalized by densitometry of α-actin (1:2000 – Dako).

### Quantitative Real-Time PCR (qPCR) Analysis

Total RNA was isolated from aortas using TRizol^®^ Reagent according to the manufacturer’s instructions. mRNAs encoding the CYP4A1 and CYP4F3 were quantified by qPCR using Fast SYBR^®^ Green Master Mix (Thermo Fisher Scientific, MA, United States). GAPDH was used as an internal control. qPCR reactions were performed, recorded, and analyzed using the Corbett Research system (Corbett Life Sciences, Sydney, Australia). The conditions for qPCR were as follows: 95°C for 20 s, 40 cycles of 95°C for 3 s and 60°C for 30 s. Cycle threshold (*C*t) values obtained for each gene were referenced to GAPDH (Δ*C*t) and converted to the linear form using the term 2^-ΔΔCt^ as a value directly proportional to the copy number of complementary DNA and initial quantity of mRNA. Primer sequences: CYP4A1 F: CGACACAGCCACTCATTCCT; R: TCAGGGCCACAATCACCTTC (97pb) (NM_175837.1); CYP4F3 F: GTGCGTCTTCAGCTTTGACAG; R: GTCCACATGCAGAAGCAGACT (118pb) (NM_001033686.1); GAPDHF: GGGCAGCCCAGAACATCAT; R: CCGTTCAGCTCTGGGATGAC (76bp) (NM_017008.4).

### Activity of CYP Enzymes and 20-HETE Concentration

Thoracic aortas were homogenized in ice-cold PBS with 2 μg/ml protease inhibitors. Total protein amount was determined by Qubit fluorometric quantitation (Thermo Fisher Scientific). Assays for activity of CYP4A1 and CYP4F3 were performed using the instructions in the P450-Glo^TM^ Assay (Promega, Madison, WI, United States). Biochemical assays were assembled and performed in opaque white 96-well plates, and reactions were incubated with specific CYP substrates 100 μM Luciferin-ME (CYP4A1) or 50 μM Luciferin-4F2/3 (CYP4F3) for 30 min at 37°C. After incubation period, 50 μl of Luciferin Detection Reagent was added to each well to terminate the reaction and initiate luminescence. Luminescence was detected every 5 min for 30 min using a Synergy multi-mode plate reader (BioTek Instruments, Winooski, VT, United States). Data was normalized by the amount of protein in each sample and expressed as the percentage (%) of luminescence emitted by recombinant human CYP4A1 or CYP43F with P450 reductase (Supersomes^TM^, BD Biosciences). In another series of experiments, the concentration of 20-HETE was determined in aortic homogenates with a commercially available ELISA kit (Detroit R&D, Detroit, MI, United States). 20-HETE levels were expressed as ng/ml and normalized by the total amount of protein.

### Statistical Analysis

Data were expressed as means ± SEM (Standard Error of the Median). The number (*n*) of rats used in each experiment was indicated in the figure legends. Phenyl vasoconstriction was expressed as delta of contraction subtracted from the initial tension. Area under curve (AUC) was calculated from each individual concentration-response curve and expressed as arbitrary units. Differences among groups (SHAM, OVX, CEE and CEE+T) were analyzed by one-way ANOVA with Tukey *post hoc* test. Statistical significance was accepted at *p* < 0.05. The statistical analysis was carried out using the Prism 5 software (GraphPad Software Inc., San Diego, CA, United States).

## Results

### Uterine Weight, Sex Steroid Hormone Levels and Arterial Blood Pressure

**FIGURE 1:**
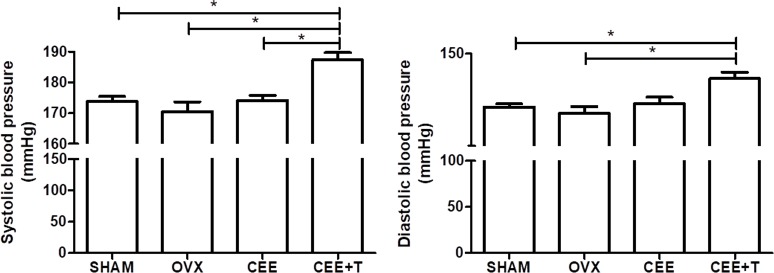
Systolic and diastolic arterial blood pressure (mmHg) were obtained from SHAM, ovariectomized (OVX), OVX treated with conjugated equine estrogens (CEE) and OVX treated with CEE plus testosterone (CEE+T) female spontaneously hypertensive rats. Results represent the mean ± SEM from 6 to 8 animals/group. One-way ANOVA: ^∗^*P* < 0.05.

Ovariectomy reduced significantly uterine weight and plasma estrogen level when compared to the SHAM female rats and were restored by CEE and CEE+T treatments. Testosterone level was increased significantly in the CEE+T group compared to all groups (**Table 1**). The systolic and diastolic arterial pressures of female SHR were neither modified by ovariectomy nor by CEE treatment. However, the association of testosterone with CEE markedly increased systolic arterial pressure compared to the SHAM, OVX, and CEE groups and increased diastolic arterial pressure compared to the SHAM and OVX groups (**Figure 1**).

**Table 1:** Uterine weight and sex steroid hormone levels in SHAM, ovariectomized (OVX), OVX treated with conjugated equine estrogens (CEE) and OVX treated with CEE plus testosterone (CEE+T) female spontaneously hypertensive rats.

	Sham	ovx	CEE	CEE+T
Wet uterus weight (g/cm tibia)	0.117 α 0.01	0.016 α 0.1*	0.086.3 α 0.1#	0.085 α 0.1#
Dry Uterus weight (g/cm tibia)	0.036 α 0.01	0.007 α 0.01*	0.024 α 0.01#	0.033 α 0.01#
Plasma estrogen (pg/ml)	37.8 α 3.1	13.7 α 1.2*	74.0 α 13.3*#	41.8 α 7.1*#
Plasma testosterone (ng/ml)	13.6 α 4.4	6.5 α 0.4	6.5 α 0.5	29.7 α 6.1*#+

*Results are expressed as mean ± SEM of 5–6 animals/group. One-way ANOVA: ^∗^P < 0.05 versus SHAM; ^#^P < 0.05 versus OVX; ^+^P < 0.05 versus CEE.*

### Vascular Reactivity

**FIGURE 2:**
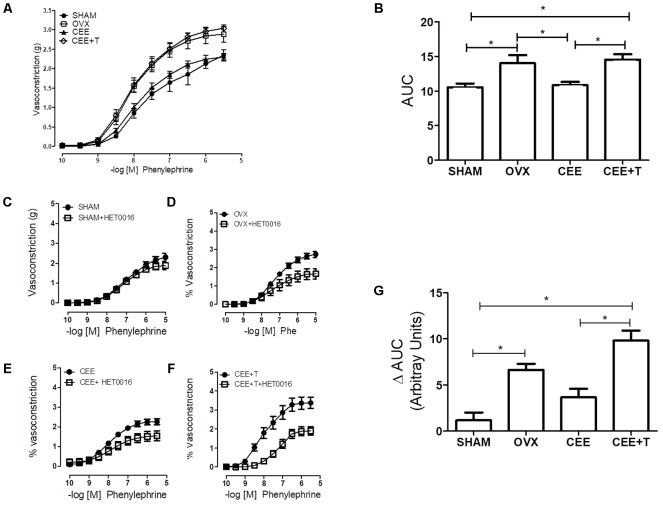
Cumulative Concentration-Response Curves (CCRC) to phenylephrine (Phe) **(A)** and area under curve (AUC) to Phenyl **(B)**, in endothelium-intact aorta from SHAM, ovariectomized (OVX), OVX treated with conjugated equine estrogens (CEE) and OVX treated with CEE plus testosterone (CEE+T) female spontaneously hypertensive rats. CCRC to Phenyl in the presence of HET0016, the CYP4A and CYP4F inhibitor, in aorta from SHAM **(C)**, OVX **(D)**, CEE **(E)**, and CEE+T **(F)** rats. Difference of AUC in the absence and presence of HET0016 **(G)**. Results represent the mean ± SEM from 6 to 8 independent experiments. ^∗^*P* < 0.05.

Phenyl contracted thoracic aortic rings in a concentration-dependent manner (**Figure 2A**). Contractions induced by Phenyl were increased in aortic rings from OVX group in comparison to SHAM group (**Figure 2B**). CEE treatment reduced the Phenyl-contractile response in aorta isolated from OVX-SHR, restoring the vascular responses to similar levels of aortas from SHAM rats. However, the protective effects of CEE were abolished when testosterone was associated to CEE treatment in OVX-SHR (**Figure 2B**).

In order to evaluate the contribution of 20-HETE to the increased vasoconstriction observed in OVX and CEE+T groups, Phenyl contractions were performed in the presence of HET0016 (1 μM), a CYP4A and CYP4F inhibitor. Addition of HET0016 to the organ bath did not interfere with Phenyl-induced vasoconstriction in SHAM aortic rings (**Figure 2C**). On the other hand, inhibition of CYP4A and CYP4F by HET0016 reduced Phenyl-induced constriction in aorta isolated from OVX, CEE, and CEE+T groups, respectively (**Figures 2D–F**). The delta analysis of these effects showed that HET0016-induced reduction in Phenyl contractions were greater in the OVX compared to the SHAM group and did not change in the CEE group. In contrast, the decrease in Phenyl contractions, induced by HET0016, was greater in CEE+T than SHAM and CEE groups (**Figure 2G**).

### ROS Detection

**FIGURE 3:**
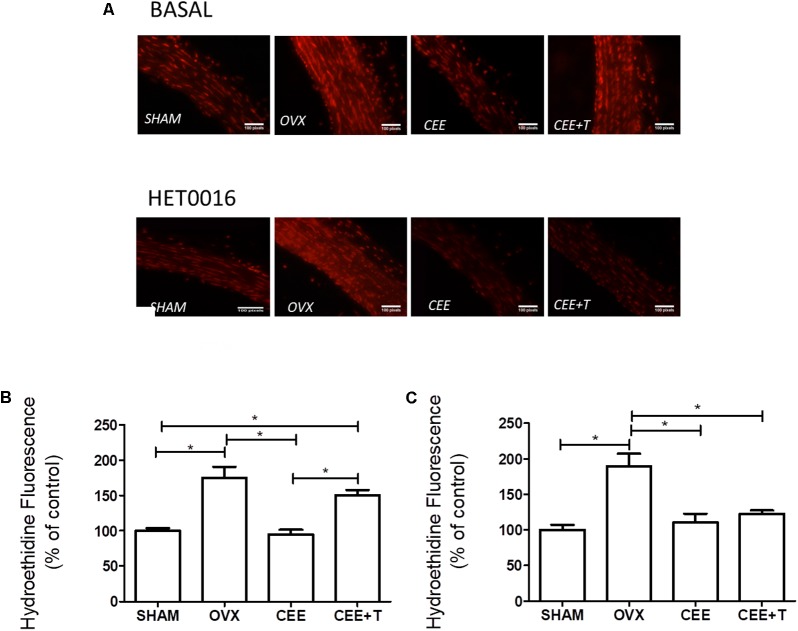
Representative fluorescent images **(A)** of nuclei labeled with ethidium produced by oxidation of dihydroethidium (DHE) by reactive oxygen species in aortic sections of SHAM, ovariectomized (OVX), OVX treated with conjugated equine estrogens (CEE) and OVX treated with CEE plus testosterone (CEE+T) female spontaneously hypertensive rats. Bar graphs show the densitometric analyses of fluorescence intensity of arteries after treatment with vehicle **(B)** and 1 μM of HET0016, the CYP4A and CYP4F inhibitor **(C)**. Results were expressed as percentage of SHAM and represent the mean ± SEM from 6–8 independent experiments. One-way ANOVA: ^∗^*P* < 0.05.

An increase in ROS levels was detected in aorta isolated from OVX compared to aorta of the SHAM group (**Figure 3A**). CEE treatment reduced ROS production in aortas of OVX to similar level observed in aorta of the SHAM group. Nevertheless, the association of testosterone to CEE abolishes this effect of CEE, maintaining the levels of ROS similar to those observed in OVX (**Figure 3B**). Treatment of aortas with HET0016 (1 μM), a CYP4A and CYP4F inhibitor, reduced ROS generation only in aorta isolated from the CEE+T group (**Figure 3C**).

### CYP4A1 and CYP4F3 Protein and mRNA Expression

**FIGURE 4:**
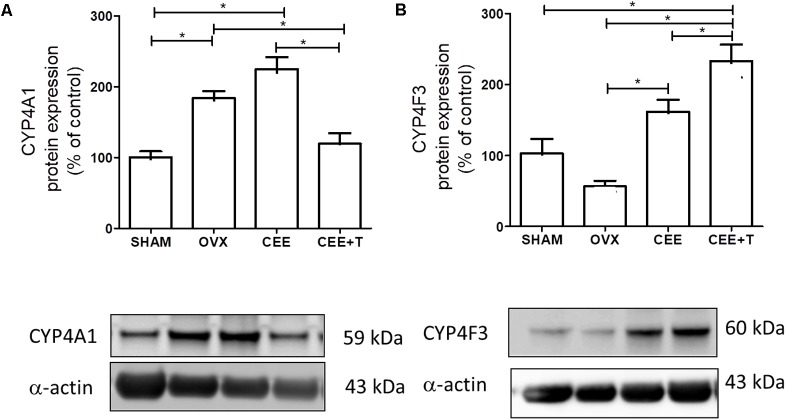
Immunoblot representative images (bottom) and densitometric analyses (top) of protein expression of CYP4A1 **(A)** and CYP4F3 **(B)**, normalized by α-actin, in aorta of SHAM, ovariectomized (OVX), OVX treated with conjugated equine estrogens (CEE) and OVX treated with CEE plus testosterone (CEE+T) female spontaneously hypertensive rats. Results were expressed as percentage of SHAM and represent the mean ± SEM from 6 to 7 independent experiments. One-way ANOVA: ^∗^*P* < 0.05.

**FIGURE 5:**
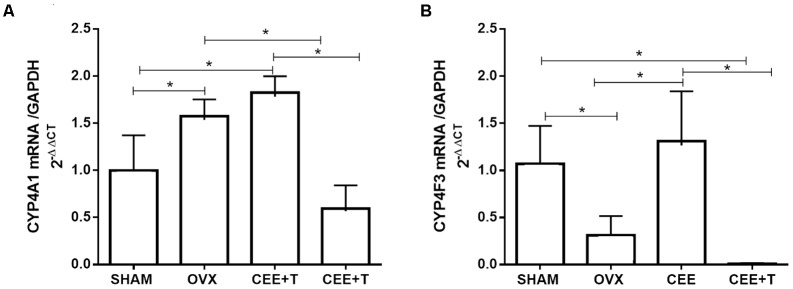
mRNA levels of CYP4A1 **(A)** and CYP4F3 **(B)** normalized by GAPDH in aorta of SHAM, ovariectomized (OVX), OVX treated with conjugated equine estrogens (CEE) and OVX treated with CEE plus testosterone (CEE+T) female spontaneously hypertensive rats. Results represent the mean ± SEM from 3 to 6 independent experiments. One-way ANOVA: ^∗^*P* < 0.05.

Ovariectomy increases protein expression of CYP4A1 in aorta of SHR. CEE+T, but not CEE alone, decreases CYP4A1 expression compared to OVX to the similar level of the SHAM group (**Figure 4A**). On the other hand, in aorta of OVX the CYP4F3 protein expression level were similar to the ones observed in SHAM. CYP4F3 expression was augmented by CEE treatment in comparison to OVX group, and further increased by CEE+T (**Figure 4B**). mRNA levels of CYP4A1 follow the same pattern of protein expression in SHR aortas (**Figure 5A**). However, mRNA levels of CYP4F3 in the aorta of OVX are marked decreased compared to the levels observed in SHAM. The CEE treatment augmented the CYP4F3 in OVX group, while the association of testosterone to CEE treatment decreased CYP4F3 mRNA levels above the levels observed in OVX (**Figure 5B**).

### CYP4A1 and CYP4F3 Activity and 20-HETE Concentration in Rat Aorta

**FIGURE 6:**
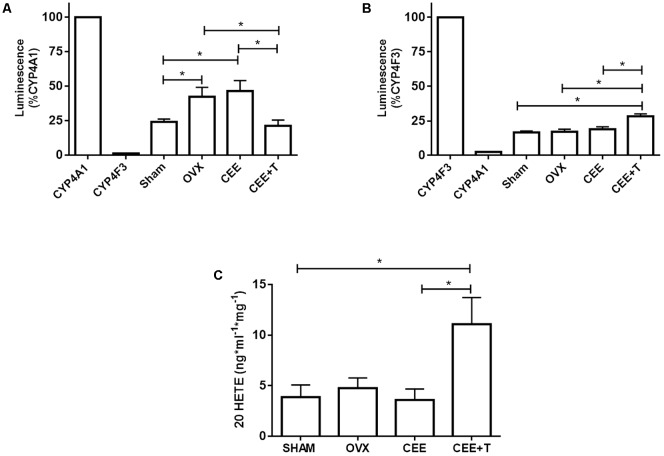
Luminescence induced by CYP4A1 **(A)** and CYP4F3 **(B)** substrates, and **(C)** 20 HETE levels in aorta of SHAM, ovariectomized (OVX), OVX treated with conjugated equine estrogens (CEE) and OVX treated with CEE plus testosterone (CEE+T) female spontaneously hypertensive rats. Enzyme activity is expressed in percentage relative to the luminescence induced by recombinant CYP4A1 or CYP4F3 enzymes. Results represent the mean ± SEM from 3 to 6 independent experiments performed in duplicate. One-way ANOVA: ^∗^*P* < 0.05.

Luminescent signals for Luciferin-ME or Luciferin-4F2/3 was specific for recombinant CYP4A1 or CYP4F3, respectively (**Figures 6A,B**), and completely abolished (∼90% decrease) in the presence of 1 μM HET0016 (data not shown). The activity of CYP4A1 was increased in aortic homogenate of OVX compared to SHAM. CEE treatment did not modify CYP4A1 activity in OVX, although the association of CEE with testosterone markedly decreased CYP4A1 activity (**Figure 6A**). As for CYP4F3 activity, only aortas from OVX treated with CEE+T presented a significant increase in relation to the other groups (**Figure 6B**). The increase in CYP4F3 activity was paralleled with an increase of 20-HETE levels in aortic tissue (**Figure 6C**).

## Discussion

The benefits of treatment with testosterone in HSDD have been known for a long time (Palacios, 2011) and strengthened by randomized controlled data supporting its use (Woodis et al., 2012). However, important unanswered questions in the cardiovascular system still exist.

**FIGURE 7:**
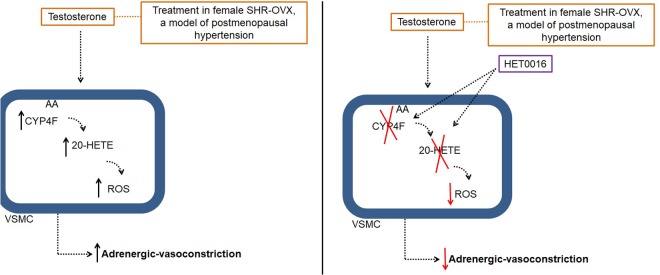
Illustrative image of testosterone effects in aorta of ovariectomized (OVX) Spontaneously Hypertensive Rat (SHR), a model of postmenopausal hypertension. AA: acid arachidonic; 20-HETE: 20-hydroxyeicosatetraenoic acid; HET0016: *N*-hydroxy-*N*′-(4-n-butyl-2-methylphenyl)Formamidine; ROS, reactive oxygen species; VSMC, vascular smooth muscle cells.

In this study, we demonstrated that association of a clinically relevant dose of testosterone to CEE therapy induces Phenyl-hyperreactivity and increases ROS generation by a mechanism involving CYP4F3-derived 20-HETE synthesis (**Figure 7**). Those findings added important mechanistic information to previous study showing that the association of testosterone to CEE abolishes the vascular protective effect of estrogens in aorta of ovariectomized SHR, a model of postmenopausal hypertension (Costa et al., 2015).

Elevated androgen/testosterone levels have been linked to the development of hypertension (Lopes et al., 2012; Tostes et al., 2015), therefore we hypothesized that treating hypertensive females with testosterone could interfere with blood pressure levels, worsening hypertension. We found that ovariectomy and CEE treatment does not alter systolic and diastolic blood pressure compared to that observed in intact (SHAM) hypertensive female rats. Nevertheless, the association of testosterone to estrogen therapy markedly increased blood pressure in hypertensive OVX females, confirming previous data of our group (Costa et al., 2015). Besides, hormone-mediated and sex-associated differences in control of blood pressure has been reported in several experimental models of hypertension (Reckelhoff et al., 1998; Reckelhoff and Granger, 1999; Reckelhoff, 2001) and a significant correlation between androgen levels and the development of hypertension and other cardiovascular disease has been well reviewed by Liu et al. (2003).

Androgen receptors are widely distributed in the vascular tissues, both in the vascular endothelium and smooth muscle cells (Lopes et al., 2012), and therefore testosterone could modulate arterial pressure by a direct modulation of vascular function. Testosterone has been associated with potentiation of vasoconstriction elicited by angiotensin II (Chen et al., 1992; Reckelhoff et al., 2000; Sartori-Valinotti et al., 2008; Yanes et al., 2009), increased thromboxane production by cyclooxygenase (Song et al., 2004; Gonzales et al., 2005) and Cyp4A/20-HETE activation (Holla et al., 2001; Skott, 2003; Singh and Schwartzman, 2008; Lopes et al., 2012). In fact, the role of 20-HETE inhibition by HET0016 in blood pressure has been described. Yanes et al. (2011) demonstrated that in aged female spontaneously hypertensive, as a model of postmenopausal hypertension, acute intravenous infusion of HET0016 also reduced blood pressure and Toth et al. (2013) demonstrate that 5 days of treatment with HET0016 can reduce blood pressure in male spontaneously hypertensive rats.

We demonstrate that association of testosterone to estrogen therapy abolished the beneficial effects of CEE treatment in aortas of OVX-SHR, by increasing adrenergic vasoconstriction. Supporting our data are previous studies showing that testosterone enhances vasoconstriction responses to different agonists (Khalil, 2005; Chinnathambi et al., 2014a,b), even when it is associated with estrogen therapy (Costa et al., 2015).

The arachidonic acid ω-hydroxylase metabolite 20-HETE promotes vasoconstriction and increases vascular resistance (Alonso-Galicia et al., 1999; Miyata and Roman, 2005), and previous studies have established 20-HETE as an important mediator in the vasoconstriction induced by endothelin (Hercule and Oyekan, 2000) and angiotensin II (Croft et al., 2000; Joly et al., 2006). We observed that the hyperactivity to Phenyl, when OVX-SHR where treated with the association CEE+T, was partially mediated by 20-HETE, since acute inhibition of aortas with HET016 markedly decreased Phenyl contractions (Δ AUC).

The HET0016 is an inhibitor of 20-HETE formation. (Miyata et al., 2001) have shown that the HET0016 potently and selectively inhibited the production of 20-HETE in microsomal renal by inhibited different CYP isoforms. We utilized the concentration similar to (Yousif and Benter, 2010), because that is efficient for decrease phenylephrine induced-vasoconstriction in microvessels of SHR and Wistar Kyoto.

Many factors may contribute to changes in 20-HETE concentration in the vasculature, among them are sex hormones (Coon, 2005). Moreover, in non-vascular tissues up-regulation of CYP4A expression by estrogen has been also observed (Hiratsuka et al., 1996; Zhang and Klaassen, 2013). Holla et al. (2001) have demonstrated that androgen-mediated regulation of CYP4A arachidonate monooxygenases are important to control systemic blood pressure. Berezan et al. (2008) demonstrated that ovariectomy in aged normotensive Sprague–Dawley rats increases Phenyl-induced vasoconstriction via up-regulation of CYP4A expression, an effect that was not reversed by estrogen treatment. In the present study, we found similar results on CYP4A1 expression and activity, with CYP4A1 being augmented in OVX-SHR and not modified by CEE.

There is evidence that increased vascular ROS generation promoted by testosterone is associated with endothelial dysfunction (Costa et al., 2015), contributing to changes in blood pressure. In addition, an increase in ROS levels by androgens has been reported in vascular smooth muscle cells of Wistar and SHR male rats (Caplice et al., 2003; Chignalia et al., 2012). In the present study, we showed that adding testosterone to CEE therapy increases aortic ROS generation, abolishing the vascular protective effects exerted by estrogen. As established by our group, the increase of ROS levels in the vasculature of OVX-SHR is mostly associated to the up-regulation of NADPH oxidase subunits (Dantas et al., 2004; Costa et al., 2015) and reduced antioxidants enzymes (Ceravolo et al., 2012). In our previous study, we interestingly observe that both CEE and CEE+T equally inhibit the up-regulation of NADPH oxidase subunits NOX2 and p22-phox in aortas of OVX-SHR, and therefore this mechanism could not account for the increase in ROS production on CEE+T-treated females SHR. In this regard, we proposed that testosterone increases ROS generation by increasing NADPH activity instead of modulating its expression (Costa et al., 2015).

As endothelial CYP450 activity and expression can be stimulated by hormones (Popp et al., 1998), and considering our finding that 20-HETE modulates vasoconstriction in CEE+T-treated OVX-SHR, we hypothesized that this pathway could contribute to activate NADPH oxidase and increase ROS generation. In fact, 20-HETE can activate NADPH oxidase promoting ROS generation (Medhora et al., 2008; Toth et al., 2013). Also, the increase of CYP-induced 20-HETE or testosterone levels have been previously reported to induce ROS production in blood vessels (Cheng et al., 2008; Medhora et al., 2008; Costa et al., 2015). We observed that inhibition of 20-HETE production by HET0016 decreased ROS generation elicited by testosterone treatment. In the same way, HET0016 promotes anti-oxidative effects directly in the cerebral microvasculature (Toth et al., 2013). In cerebral circulation, the higher level of 20-HETE promotes inflammation through ROS production (Toth et al., 2013).

Different CYP isoforms have been described to generate varying amounts of ROS in different tissues (Puntarulo and Cederbaum, 1998). For example, CYP2C9 generates ROS in coronary arteries (Fleming, 2001) and likewise vascular dysfunction in Dahl salt-sensitive hypertensive rat seems to be due to 20-HETE-mediated ROS production (Williams et al., 2010; Lukaszewicz and Lombard, 2013). In endothelial cells, CYP4F2 overexpression increases 20-HETE and superoxide anion production by activation of NADPH oxidase (Cheng et al., 2014). Also in the endothelium, CYP2C contributes to the stretch-induced generation of anion superoxide which has until now been attributed to the activation of the NADPH oxidase (Hishikawa and Lüscher, 1997; Hishikawa et al., 1997). The present study was the first time to demonstrated that vascular dysfunction and ROS generation in may also involve CYP4F3-derived products.

Several studies have showed that there is an association among androgen, CYP4A and CYP4F expression, 20-HETE synthesis and hypertension (Holla et al., 2001; Nakagawa et al., 2003; Zhou et al., 2005; Wu et al., 2013; Cheng et al., 2014). The knowledge of sex hormones contribution to CYP4A and 4F expression have improved our understanding of how these enzymes are regulated by circulating hormone levels (Zhang and Klaassen, 2013). For example, reduced plasmatic levels of testosterone caused by castration, decreases mRNA expression of CYP4A12A and 12B in liver and kidney. Similar effect was observed in female-OVX treated with dihydrotestosterone (Zhang and Klaassen, 2013). We demonstrated that ovariectomy in SHR increases CYP4A1 activity up-regulating its mRNA and protein expression. Curiously, estrogen therapy used in the present study did not modify CYP4A1 activity and expression, although the association of testosterone to CEE markedly reduced CYP4A1.

On the other hand, protein expression of CYP4F3 was augmented by estrogen therapy in OVX-SHR and potentiated when testosterone added to CEE. This further increase of CYP4F3 expression in CEE+T was associated with an increase in CYP4F3 activity and 20-HETE and paralleled to the increased ROS and Phenyl contraction observed in CEE+T rats. The CYP4F enzyme is another subfamily of CYP that contributes to 20-HETE synthesis (Harmon et al., 2006; Wu et al., 2014). A clinical study in Chinese cohorts have shown a correlation of hypertension in patients with *CYP4F* polymorphisms and elevated levels of urinary 20-HETE (Liu et al., 2008; Deng et al., 2010). Similarly, estrogen regulated CYP4F1, F4 and F6 expression (Kalsotra et al., 2002) and androgen treatment induces CYP4F2 expression and activity, leading to an enhance in urinary 20-HETE production (Liu et al., 2012).

The most intriguing point in this study was the observation that change in hormone milieu (by OVX), estrogen and the association of estrogen with testosterone have distinct effects in different enzymes of CYP family, and that the variance in the modulation of CYP4A and CYP4F may promote different effects on vasoconstriction and ROS production. The cascade of arachidonic acid (AA) is very complex and comprises a myriad of enzymes which the tuning among them is still unknown. When it goes to the branch of the Cytochrome P450 superfamily, more complexity and lack of knowledge is added. Both CYP4A and CYP4F are ω-hydroxylases that metabolize AA into several hydroxy-eicosatetraenoic acids (HETEs), having many of them vasoconstrictor properties (Roman, 2002; Nebert et al., 2013). Although CYP4A1 has been described with the highest catalytic efficiency to convert AA into 20-HETE, in our model (aorta of female SHR) we found a direct association of increased 20-HETE with an increase of CYP4F3 activity/expression. Our hypothesis is that the increase of 20-HETE via CYP4F3 could account for ROS generation in OVX-SHR treated with CEE+T, while the increase of CYP4A1 in OVX and OVX treated with CEE could favor the production of other HETEs that could contribute to Phenyl contraction.

## Conclusion

Our data confirms that association of testosterone to CEE treated- OVX-SHR, a postmenopausal hypertensive model, rises systolic and diastolic blood pressure and increases adrenergic vasoconstriction and ROS generation in isolated aorta. The great novelty of this study describes the contribution of CYP4F3-derived synthesis of 20-HETE to the detrimental effects of CEE+T therapy. Our studies call the scientific community for the need of clinical studies to improve our knowledge on the cardiovascular effects of the association of testosterone to conventional hormone therapy in HSDD postmenopausal women. Besides, our findings provide a rational that cytochrome p-450 pathways/20-HETE synthesis may be an important therapeutic target for prevention/treatment of cardiovascular diseases in women in the presence of high levels of testosterone.

## Author Contributions

TC, GC, EA, AD, and MC: conception or design of the work. TC, GC, CE, CH, BC, MO, RS-E, FJ-A, EA, AD, and MC: performed the experiments, acquisition, analysis, and interpretation of data for the work. TC, GC, CE, FJ-A, EA, AD, and MC: drafting the work or revising it critically for important intellectual content. TC and MC: final approval of the version to be published.

## Conflict of Interest Statement

The authors declare that the research was conducted in the absence of any commercial or financial relationships that could be construed as a potential conflict of interest.
